# A microRNA with a mega impact on plant growth: miR156ab spray keeps drought away

**DOI:** 10.1093/plphys/kiad161

**Published:** 2023-03-14

**Authors:** Alaeddine Safi

**Affiliations:** Plant Physiology, American Society of Plant Biologists, USA; Department of Plant Biotechnology and Bioinformatics, Ghent University, Ghent B-9052, Belgium; VIB Center for Plant Systems Biology, Ghent B-9052, Belgium

Domestication by humans has adapted wild plants into cultivable crops. Through this long process of recurrent selections, modern crop genomes have been reshaped in favor of a number of desirable characteristics (e.g. yield, taste, texture, and flesh-to-seed ratio). The prioritization of those traits may have indirectly resulted in the loss of others. Indeed, the unprecedented rate of climate change has uncovered the vulnerability of our modern crops to extreme weather (Hirt et al. 2023). For example, the modern cultivated apple (*Malus domestica*) is more sensitive to drought than its wild ancestor (*Malus sieversii*) (Liao et al. 2017). Apple is 1 of the world’s most widely cultivated and economically important fruits. Yet the increasingly frequent and intense drought episodes threaten not only fruit productivity and quality in mature plants but also plant growth at early stages (Sun et al. 2022). Thus, improving drought tolerance is a pressing necessity.

In this issue of *Plant Physiology*, Feng et al. (2023) investigated the role of a drought-responsive microRNA (miR156ab) in apple drought tolerance. Overexpressing Ms-miR156ab (from the tolerant ancestor plant) in a domesticated cultivar (GL-3) or in Arabidopsis (*Arabidopsis thaliana*) substantially enhanced plant growth. The transgenic lines formed not only more and longer roots compared with GL-3 but also taller shoots and larger leaves under normal growth conditions (Figure). Gene expression analysis on the overexpressor plants (Ms-miR156ab_OE) showed that genes involved in auxin biosynthesis (*MdYUCCAs*), transport (PIN-FORMED carriers: *MdPINs*), and signaling (LOB domain-containing protein 18: *MdLBD18*) were upregulated, whereas auxin conjugation genes (GRETCHEN HAGEN 3: *MdGH3s*) were downregulated. These changes in gene expression are translated into auxin content increase, which in turn promotes plant growth. More interestingly, both Arabidopsis and apple overexpressor lines were also more tolerant of simulated drought conditions (Figure).

**Figure.**
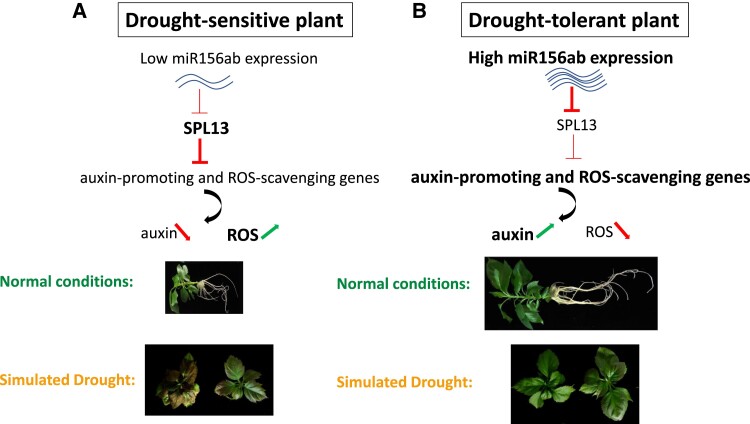
miR156ab is crucial for plant growth and drought tolerance. **A)** In drought-sensitive plants, high SPL13 expression inhibits auxin-promoting and ROS-scavenging genes leading to less auxin and elevated ROS contents. Consequently, SPL13 attenuates plant growth and tolerance to abiotic stress. **B)** The higher miR156ab expression in drought-tolerant plants reduces the abundance of SPL13 and thus releases the repression of auxin-promoting and ROS-scavenging genes. The elevated auxin levels together with the reduced oxidative stress help the plant to thrive and tolerate unfavorable growth conditions such as drought. Adapted from Feng et al. (2022).

miR156-SQUAMOSA PROMOTER-BINDING PROTEIN-LIKE (SPL) is a conserved module involved in various pathways including auxin signaling, root development, and drought tolerance (Arshad et al. 2017; Dai et al. 2018). Thus, the authors studied the SPL transcription factor (TF) family in *M. domestica* and found that among the 32 identified members, *MdSPL13* and *MdSPL26* were strongly downregulated in Ms-miR156ab_OE lines. Using degradome sequencing and 5′ rapid amplification of cDNA ends (5′-RACE), they proved that indeed Ms-miR156ab cleaves both *SPL* mRNAs at the conserved target sites. For further confirmation, they pasted *MsSPL13* or *MsSPL26* cleavage sites to the *luciferase* coding sequence and found that the luciferase signal declined considerably when transiently coexpressed with Ms-miR156ab in *Nicotiana benthamiana* leaves. No decrease in signal was noticed when the cut sites were replaced by mutated ones or by a random sequence. Jointly, these data demonstrate that *MsSPL13* or *MsSPL26* expression levels are directly controlled by Ms-miR156ab, suggesting they are also involved in growth enhancement and drought tolerance. Indeed, Arabidopsis plants overexpressing *MsSPL13* (MsSPL13_OE) were affected in shoot and root growth and were more susceptible to drought stress (Feng et al. 2023). The same plants also showed downregulation of *AtYUCCA6* and *AtPIN1* and upregulation of *AtGH3-1*, which explained the decrease in auxin content.

Yeast 1-hybrid and electrophoretic mobility shift assay (EMSA) experiments indicated that MsSPL13 and/or MsSPL26 bind at least 1 region in auxin-related gene promoters. In agreement with the reverse transcription quantitative PCR (RT-qPCR) results, luciferase transactivation assay revealed that MsSPL13 and MsSPL26 induce *MsYUCCAs*, *MsPINs*, and *MsLBD18* expression and repress that of *MsGH3s*. Together, these results establish that MsSPL13 and/or MsSPL26 TFs directly regulate the expression of those genes to repress auxin-dependent plant growth.

Interestingly, MsSPL13 was also able to bind the promoter and inhibit the expression of *Responsive to Desiccation 22* (*RD22)* and *SuperOxide Dismutase* (*SOD*) genes encoding for reactive oxygen species (ROS)-scavenging enzymes. In addition to its limited expression, the activities of SOD and other ROS-scavenging enzymes declined in MsSPL13_OE lines under both normal and stress conditions, leading to higher ROS levels. Conversely, Ms-miR156ab_OE plants revealed opposite behavior marked by higher SOD and catalase (CAT) activities and lower hydrogen peroxide (H_2_O_2_) and superoxide anion (O_2_^·–^) accumulation. Those lines had lower levels of malondialdehyde (oxidative stress marker) and higher levels of proline (involved in abiotic stress protection) (Liang et al. 2013). These findings largely explain the enhanced drought tolerance in Ms-miR156ab_OE plants (Figure).

In summary, Feng et al. (2023) illustrate that MsSPL13 is a negative regulator of plant growth and drought tolerance by directly inhibiting auxin-promoting and ROS-scavenging genes. As a response to drought stress, Ms-miR156ab is induced to degrade *MsSPL13* mRNA and mitigate its repressing activity, allowing the plant to thrive and cope with this stress (Figure). By comparing the drought-tolerant apple plant (*M. sieversii*) and a drought-susceptible 1 (*M. domestica*), the authors found that these species share exactly the same core sequence of miR156ab. However, few differences in their respective promoter sequences lead to differential basal transcription activities and drought responsiveness. Thus, the drought resistance in *M. sieversii* is, at least in part, due to the higher miR156ab expression level.

It is noteworthy that miR156ab is also involved in diverse aspects of plant development as well as different biotic and abiotic stresses in various plant species (Cui et al. 2014; Zhang et al. 2017). These data provide valuable insights that could be exploited in plant breeding programs to develop more resilient crops using miR156ab expression as a molecular marker. Alternatively, exogenous miR156ab treatment could be considered in the future. For example, miR156ab could be sprayed onto trees using some of the strategies being developed to boost the adherence, stability, and cellular uptake of topical-applied RNA (Mitter et al. 2017).
